# RETRACTION: Seasonal Coat Colour Moult and Mismatch in Ezo Mountain Hares (*Lepus timidus ainu*): Phenotypic Plasticity and Snow Cover Effects in Hokkaido, Japan

**DOI:** 10.1002/ece3.74655

**Published:** 2026-11-12

## Abstract

**RETRACTION**: F. Chaudhary, J. Morimoto, T. Sakiyama and R. U. Haq, “Seasonal Coat Colour Moult and Mismatch in Ezo Mountain Hares (*Lepus timidus ainu*): Phenotypic Plasticity and Snow Cover Effects in Hokkaido, Japan,” *Ecology and Evolution* 16, no. 8 (2026): e74052, https://doi.org/10.1002/ece3.74052.

The above article, published online on 18 August 2026 in Wiley Online Library (wileyonlinelibrary.com), has been retracted by agreement between the journal Editor‐in‐Chief, Allen Moore; the Executive Editor, Arley Muth; the British Ecological Society; and John Wiley & Sons Ltd. The retraction has been agreed upon following concerns raised by a third party. An investigation found that several references cited within the article were invalid or could not be verified. The authors acknowledged that multiple referencing errors had been made and proposed corrections, including the removal and replacement of affected references. However, due to the extent and nature of these errors, the Editors have lost faith in the article and therefore decided a retraction is appropriate. The authors disagree with the retraction.

**RETRACTION**: F. Chaudhary, J. Morimoto, T. Sakiyama and R. U. Haq, “Seasonal Coat Colour Moult and Mismatch in Ezo Mountain Hares (*Lepus timidus ainu*): Phenotypic Plasticity and Snow Cover Effects in Hokkaido, Japan,” *Ecology and Evolution* 16, no. 8 (2026): e74052, https://doi.org/10.1002/ece3.74052.

The above article, published online on 18 August 2026 in Wiley Online Library (wileyonlinelibrary.com), has been retracted by agreement between the journal Editor‐in‐Chief, Allen Moore; the Executive Editor, Arley Muth; the British Ecological Society; and John Wiley & Sons Ltd. The retraction has been agreed upon following concerns raised by a third party. An investigation found that several references cited within the article were invalid or could not be verified. The authors acknowledged that multiple referencing errors had been made and proposed corrections, including the removal and replacement of affected references. However, due to the extent and nature of these errors, the Editors have lost faith in the article and therefore decided a retraction is appropriate. The authors disagree with the retraction.

